# "The Hospital" Nursing Mirror

**Published:** 1901-08-24

**Authors:** 


"The Hospital" Nursing Mirror.

Being the Nursing Section of "The Hospital."

[Contributions for this Section of "The Hospital" should be addressed to the Editor, "The Hospital" Nursing Mirror, 28 & 29 Southampton Street Strand, London, W.C.]

### Notes on News from the Nursing World.

### HONOUR FOR A NURSE.

The King has been pleased to confer the Order of St. John of Jerusalem on Miss Edith Atkinson and to enrol her as an honorary serving sister of the Order, for special service volunteered during the outbreak of plague in Bombay, where she has been working for the last twelve years under Government as Lady Superintendent of the Cama Hospital.

### OUR BELGIAN SISTER.

Nurse Alice Bron sends us an interesting letter founded on her experiences in nursing the British soldier. As a foreigner, she endorses the high opinion which English nursing sisters have expressed. Nurse Bron had expected to return to Europe last month, but she informs us that, to her great satisfaction, the authorities have decided that she can continue to serve. It is evident that while she recognises the good qualities of her patients, the manner in which she discharges her duties is also appreciated.

### A STEP FORWARD IN IRELAND.

The Irish Local Government Board has issued a new order, the following portion of which applies to nurses in workhouses.

Article 4.

(*a*) The Board of Guardians shall, as soon as may be requisite, and from time to time hereafter upon the occurrence of any vacancy, appoint, subject to our approval, fit persons to perform respectively the duties specified by our rules and regulations in force at the time to be the duties of the following officers:—

1. Clerk to the guardians.
2. Medical officer of the workhouse.
3. Master of the workhouse.
4. Nurse of the workhouse.
5. Matron of the workhouse.
6. Schoolmaster of the workhouse.
7. Schoolmistress of the workhouse.
8. Porter of the workhouse.

(*c*) The following shall be the duties of the nurse of the workhouse:—

1. To bring under the special notice of the medical officer every patient as soon as possible after admission into the sick wards.
2. To be responsible for the good nursing of the sick and for the satisfactory discharge of the duties of the nursing staff, and for the carrying out of the directions of the medical officer with respect to all medicines and medical appliances.
3. To inform the medical officer without any avoidable delay of any defects that may be observed in connection with the arrangements for the care and the nursing of the sick, including their clothing and diet.
4. To send a notification in writing to the master of the workhouse whenever the condition of any patient demands that the medical officer, the chaplain, or the relatives of such patient should be sent for or communicated with.
5. To see that everything connected with the patients and the wards is kept clean and in proper condition; and also to take care that all wards are duly ventilated, warmed, and lighted.
6. To see that the food is properly distributed to the patients, and to arrange that each patient receives the special treatment ordered by the medical officer, and generally to carry out all reasonable directions of the medical officer, to whom and to the Board of Guardians only (notwithstanding anything contained in any other General Order) she shall be subordinate, save as regards the general disciplinary control of the master of the workhouse.
7. In the absence of the medical officer, to exercise general supervision and control over the nurses, wardmaids, and attendants, and to maintain proper order and discipline in the sick wards in her charge.

(*d*) The Board of Guardians shall, subject to our approval in each case, appoint such and so many "qualified nurses" to assist the "nurse of the workhouse" in the performance of her duties as above mentioned, and generally in the nursing and care of the sick in the workhouse, and also such and so many "wardsmaids" and "attendants" for the discharge of menial duties in the infirmary or hospital, as we shall from time to time think necessary.

### THE INFLUENCE OF THE PARISH.

Meetings are being held this week in Glamorganshire on behalf of the Women's Memorial to Queen Victoria, and Lady Windsor, in announcing that it is intended to form committees for each Parliamentary division to collect contributions from the women of the county towards the endowment of the Jubilee Institute for training nurses, expresses the hope that "every parish in the county" will send at least one representative to the meetings either in Swansea or Cardiff. The former took place on Wednesday, and the latter is fixed for Saturday. There is no doubt as to the importance of enlisting the sympathy of the parishes; if this can be done, the support of the towns and cities may be relied upon. But the difficulties of rousing the parishes from their lethargy are so great that the most strenuous and persistent efforts will be needed by individual members of the committees.

### A QUESTION OF DISCIPLINE.

The Belfast Board of Guardians have called upon two of the nurses in the workhouse infirmary to send in their resignations. The nurses are sisters, and in consequence of the death of a brother-in-law they applied to the assistant superintendent of the infirmary for five days' leave of absence. She refused five days, as she could not spare them without causing much inconvenience, but suggested two days. This they declined, and stated that if they did not get the leave they wanted they would resign. At a subsequent meeting of the guardians a letter received by the committee from them, stating that they went out without leave and asking if they might come in again, was read. The committee, on the strength of an assurance by the medical officer of their capacity as nurses, recommended that they should be allowed to come in, but advised that they should be brought before the board and censured. Several guardians, however, took exception to the recommendation, and urged that, in the interests of discipline in the house, the nurses should not be retained. Eventually this view was adopted by the majority. Nor can it be said that the decision was not justified by the circumstances. The assistant superintendent had not peremptorily withheld leave, and probably the concession of two days was the utmost she could make. But in any case it would be fatal to all discipline in workhouse infirmaries if nurses who were told they could not have leave took it notwithstanding, and if guardians by their action practically encouraged disobedience.

### AN OATH FOR NURSES.

According to an American paper, an oath of professional secrecy is administered to nurses in some of the States when they receive their certificates. The wording of the oath is as follows:—

"You do solemnly swear, each by whatever you hold most sacred:

"That you will be loyal to the physicians under whom you serve, as a good soldier is loyal to his officers:

"That you will be just and generous to all worthy members of your profession, aiding them when it may be in your power to do so:

"That you will live your lives and lead your profession in uprightness and honour.

"That into whatsoever house you shall enter, it shall be for the good of the sick to the utmost of your power, and that you will hold yourselves aloof from all temptation.

"That whatsoever you shall see or hear of the lives of men and women, whether they be your patients or members of their households, you will keep inviolably secret, whether you are in other households or among your own friends.

"If you accept these obligations, let each one bow the head in sign of acquiescence.

"And now, if you shall be true to your words, may prosperity and good repute be ever yours; the opposite if you shall prove yourselves forsworn."

We do not believe that the administration of this oath is at all general in any part of America. It is said to have been suggested by the feeling that nurses gossip in one home about what has happened in another. But if a nurse knows how to hold her tongue, and to respect the confidence which is necessarily reposed in her, it is clear that there is nothing to be gained by the administration of an oath; while if she cannot restrain her tendency to chatter, and has no sense of the obligations of her calling, she will lightly esteem the most solemn pledge of professional secrecy. The idea of "swearing" nurses has, in short, no merit whatever.

### CRISIS AT WALSALL INFIRMARY.

Affairs at the Walsall Workhouse Infirmary have reached a crisis. In consequence of the refusal of the Board of Guardians to comply with the reasonable request of the probationers, that the house officials should not be allowed to wear the same uniform as the infirmary nurses, the whole of the latter have sent in their resignations. The fact was announced in the following letter, which was read at the last meeting of the board:—

We, the undersigned probationer nurses, resent the decision of the board in not granting our request, namely, about our uniform. Perhaps now the discussion has gone to its limit, the board we hope will realise the great insult (as we might term it) upon the profession not only upon ourselves, but upon the profession at large, by letting the house officials wear the same uniform as the infirmary nurses. When we entered upon our profession we expected to be treated with the respect due to nurses. We therefore beg to tender our resignations, and we hope you will release us, as under the present circumstances our duties are no longer a pleasure to us.

The clerk having read the letter, the chairman of the board ruled it out of order, on the ground that, according to the articles of agreement, the probationers could not resign. To this ruling several of the guardians took exception, but the chairman absolutely declined to allow the matter to be discussed. We observe that the Walsall papers entirely approve the course the probationers have pursued, and call upon the guardians either to accept their resignations, or to set aside the absurd order of the master of the workhouse. Whatever course may now be pursued, the popularity of Walsall Infirmary as a training school must suffer.

### THE START AT HASTINGS.

It is hoped that the nurses' residence in Old London Road, Hastings, will be opened at an early date, and that the Hastings District Nursing Association will be in working order early in October. Subscriptions and donations exceeding £100 have either been paid or promised. This is a fair financial start, but the Hastings people can, if they choose, do a good deal more. An appeal is being made for offers of suitable furniture for the Home, and the committee have a right to expect a liberal response.

### THE "BEST SOLDIERS" AT THE FRONT.

At an enthusiastic meeting held in a little country town in the Lowlands of Scotland to welcome home the brave men who, having fought for their country, had returned from the front, a nurse, who happened to be present in uniform, on leaving had her hand suddenly grasped by one of the khaki-clad veterans, who exclaimed to the crowd which surrounded him, "Eh! but the sisters' were the best soldiers out there!"

### AN OBLIGING PATIENT.

A staff nurse on night duty in one of the London hospitals was much amused by the anxiety displayed by a woman in one of the private wards to give as little trouble as possible when she realised the large amount of work that had to be done during the night. "Look 'ere, nurse!" she exclaimed one evening, "there aint no cause for you to come 'oppin' in an' out of this 'ere ward to give me my medicine, when I reckon if you was to 'and over an alarm clock, I could wake up reglar and take it myself."

### THE SALISBURY HOSPITAL, RHODESIA.

The matron of this hospital asks us to state that she has received such an enormous number of applications for posts at her institution since we inserted a notice of her requirements, that she has found it quite impossible to reply to all.

### SHORT ITEMS.

The marriage of Major R. F. Ballantine, master for 23 years of the Manchester Workhouse at Crumpsall, and Miss Sarah Jane Parrish, formerly superintendent of nurses at Watford, Herts, who was trained at Crumpsall, is announced.—Miss Edith G. Benwell has been elected president, secretary, and dispenser of the Eversfield Hospital, St. Leonards-on-Sea, in succession to Miss Payne. Miss Benwell has been connected with the London Bible Women and Nurses' Mission for 13 years, and has held successively the post of district nurse, district superintendent, and lady superintendent of the Nurses' Home. She received her training at the Seamen's Hospital, Greenwich, and at the City Road Lying-in Hospital. Miss Benwell holds the Apothecaries' Hall certificate for dispensing and also that of Westminster College.—It has been decided to hand over the balance of the fund for erecting a statue of Mr. William Rathbone to the fund of the Liverpool Queen Victoria District Nursing Fund, a work in which Mr. William Rathbone has been and is so deeply interested.—Nursing Sister Margaret McLeiss, who has been ill in South Africa, was discharged from Hospital for duty in the week ending August 11th.—The handsome sum of £57 has been realised by a fête in the grounds of Prideaux Place, Padstow, on behalf of the Cornwall County Nursing Association.

### Notes of Surgical Lectures to Nurses.

Delivered at the London Temperance Hospital by Dr. W. J. Collins, M.S., B.Sc., D.P.H.Lond., Fellow of London University and of the Royal College of Surgeons. Surgeon to the London Temperance and the Royal Eye Hospitals.

### THE LOWER EXTREMITY.

Poupart's ligament, stretching from the anterior superior spine of the ilium to the spine of the pubes, divides the abdomen from the thigh. Between its inner extremity and the pubes is Gimbernat's ligament, the sharp edge of which forms an obstacle to the return of a femoral hernia. The femoral vein lies here on the inner side of the femoral artery and immediately external to the femoral ring, through which the lymphatics of the lower extremity ascend, and along which a femoral rupture may descend. The inguinal glands may enlarge in disease of genitals, anus, or lower extremity. The long saphenous vein which has travelled from the foot along the inner side of the knee here dips down to terminate its course in the femoral vein through an opening in the fascia lata called the saphenous opening; it is through this opening that the egg-like swelling of a femoral hernia makes its way to the surface after traversing the femoral canal. The saphenous vein is the commonest seat of varix, and the area it drains is liable to varicose eczema and chronic ulcers. For the upper third of the thigh the femoral artery lies in Scarpa's triangle (formed by Poupart's ligament, the sartorius and adductor longus muscles), for the middle third in Hunter's canal betwixt the adductor and extensor muscles, while in the lower third it lies in the popliteal space having perforated the great adductor muscle. A line drawn from a point midway between the anterior superior spine of the ilium and the symphysis of the pubes to a prominent tubercle (adductor) on the inner side of the lower end of the femur indicates the course of the artery in the upper two-thirds of the thigh. Next to the aorta the popliteal artery is the commonest seat of aneurism. John Hunter showed that popliteal aneurism could be cured by ligature of the femoral artery.

The great sciatic nerve is the largest nerve in the body, and supplies the skin of the leg and greater part of the foot and the muscles of the leg, foot, and back of the thigh. It may be exposed, for stretching to cure persistent sciatica, just below the gluteus maximus (buttock) between the tuberosity of the ischium and the great trochanter of the femur, rather nearer to the former. The socket of the hip-joint (acetabulum) which receives the ball head of the femur is formed by contributions from the three bones (ischium, ilium, pubes) which in infancy make up the os innominatum. In health, a line drawn from the anterior superior spine of the ilium to the tuberosity of the ischium should just touch the top of the great trochanter of the femur (Nélaton's line). In fractures of the neck of the femur, dislocations of the hip, and the later stages of hip disease this relationship is disturbed. To ascertain the presence or absence of shortening of the lower extremity measurement is usually made from the anterior superior spine of the ilium to the internal malleolus (ankle) of the tibia.

Disease of the hip joint is generally tubercular, and may commence in the pelvis (acetabulum), the synovial membrane, or the femur. The earlier signs are lameness, limitation of movement, pain, flexion of the thigh on the pelvis, and lordosis or accentuation of the lumbar spinal curve on putting the foot down. Later there is obliteration of the fold of the buttock, shortening from destruction of the head of the femur abscess, hectic fever, amyloid disease. Pain in the knee is common in disease of the hip, and is apt to mislead. In the earlier stages the treatment consists in rest in a Thomas' splint (patten on the foot of the sound side) or plaster spica encasing hip, knee and ankle. In a later stage excision of the head of the bone and removal of all diseased tissue is practised; shortening ensues and a false joint is established. Fracture of the neck of the femur (intra-capsular) is common in old persons as the result of slight injury or indirect violence; it never unites by bone; prolonged rest in bed in such cases is therefore undesirable, as liable to induce lung or bladder trouble or bed-sores.

The knee is the largest joint in the body; it is a hinge-joint and is composed by the femur, tibia, and patella. The latter is often fractured as the result of muscular action by careful coaptation, fixation of the lower fragment, sustained traction on the upper fragment and flexion of the thigh, very good (often bony) union may be obtained. Tubercular disease of the knee-joint is very tedious and painful; the leg is displaced backwards by action of the hamstring muscles and flexed: a McIntyre splint is usually employed to correct this. Later excision (arthrectomy) may be required with a view to remove the disease and secure a stiff knee. Knock-knee (genu-valgum) is often treated by division of the femur above the knee-joint. Section of the leg one inch below the knee entails the section of one large artery (popliteal), two inches below the knee of two large arteries (anterior and posterior tibials), three inches below the knee of three large arteries (the two tibials and the peroneal).

Club-foot (talipes) may require for its cure the division of one or more of the numerous tendons which surround the ankle-joint, *e.g.*, the tendon of Achilles for talipes equinus, the two tibial tendons for talipes varu. The divided ends reunite by organising lymph, while the deformity is corrected by appropriate splints. A corn is a localised overgrowth of the horny layer of the epidermis with a tendency to extend downwards in the centre On the sole of the foot corns may ulcerate, and in cases of locomotor ataxy are apt to perforate deeply into the tissues of the foot. A bunion is an inflammatory condition of the bursa which overlies the metatarso-phalangeal joint of the great toe, and may extend so as to involve the joint. This joint is a favourite seat of gout, and is also liable to a deformity known as hallux valgus, in which there is outward deviation of the great toe; in its worst forms this requires operation to effect a cure. Tubercular disease of the bones of the foot (tarsus) and ankle is common. In the later stage Syme's amputation is resorted to, whereby the foot and lower ends of the tibia and fibula are removed and a sound flap of heel skin is preserved for walking on. Flat-foot implies the falling of the natural arches of the foot, due partly to yielding of the plantar ligaments, and largely to muscular debility, especially in persons carrying heavy loads or as the result of wearing high heels. Exostosis is not uncommon on the last phalanx of great toe: ingrowing toe-nail is also common here, requiring removal and excision of the adjacent over-growing skin.

### To Nurses.

We invite contributions from any of our readers, and shall be glad to pay for "Notes on News from the Nursing World," or for articles describing nursing experiences, or dealing with any nursing question from an original point of view. The minimum payment for contributions is 5s., but we welcome interesting contributions of a column, or a page, in length. It may be added that notices of entertainments, presentations, and deaths are not paid for, but, of course, we are always glad to receive them. All rejected manuscripts are returned in due course, and all payments for manuscripts used are made as early as possible after the beginning of each quarter.

### The Local Government Board and the Workhouse Infirmary Nursing Association.

THE President of the Local Government Board did not send the deputation from the Workhouse Infirmary Nursing Association empty away, when he received them privately at the House of Commons the other day. Mr. S. B. Provis, C.B., Permanent Secretary to the Local Government Board, Mr. W. E. Knollys, C.B., Assistant Secretary, and Dr. A. H. Downes, Poor Law Medical Inspector, were also present. The object of the deputation, which was introduced by Mr. J. G. Talbot, M.P., was to discuss the details of a statement submitted to Mr. Walter Long early in the year concerning the present condition of the nursing in country workhouse infirmaries, and the dearth of nurses obtainable for them.

### The Work of the Association.

The facts brought before the President of the Local Government Board by the deputation, which consisted of the Hon. Mrs. J. G. Talbot, Vice-President of the Executive Committee, Viscountess Knutsford, Miss Wilson, treasurer, and Miss Gill, secretary, were of great interest. It was pointed out that for over twenty-one years the attention of the Committee has been given to questions affecting the sick poor in workhouse infirmaries, and that to ensure the introduction of at least a proportion of duly qualified nurses into these institutions, the Association, during the first 18 years of its existence, had expended nearly £4,000 on the training of some 400 nurses for the Poor Law service; that these nurses were bound by agreement to work for a specified number of years in workhouse infirmaries only; and that by means of gratuities which supplemented their salaries, and of other inducements, a number of nurses otherwise trained were encouraged to accept and retain posts in infirmaries. The Association found that the initial difficulty was the reluctance of guardians to employ qualified women as nurses. With time, however, this obstacle disappeared, and the demand for their nurses became in excess of the number their funds enabled them to train. In 1895 they could only supply 85 nurses in response to a request from guardians for 199. In August 1897 the order on nursing was issued by the Local Government Board, and confirmed their opinion that the supply of trained nurses for Poor Law service had become too large a question to be dealt with by a voluntary association. They therefore decided on discontinuing the work of training nurses.

### The Results of the Order.

During the three years which have elapsed since 1897 the Association have closely watched the working of the order in country workhouse infirmaries, and the conclusions forced upon them by the facts are as follows:—In their opinion the order has, to a great extent, failed in providing a uniform standard of nursing for country workhouse infirmaries. The difficulty of obtaining and retaining trained women has increased. Persons of the pauper class are still largely employed in the capacity of nurses. The lack of trained nurses makes it easy, and in some cases furnishes an argument to the Boards of Guardians, to ignore the clause dealing with this matter. Owing to the scarcity of trained nurses available, also to a false idea of economy, guardians engage young women as probationers in situations where the means of training do not exist; a system alike injurious to the interests of patients and of probationers. The order only insists upon the superintendent nurse being properly trained. The trained superintendent is, according to the order, under the control of the workhouse master and matron in certain important departments of her work. The Association contend, in short, that it is a well-attested fact that the efficient nursing in workhouse infirmaries is hampered by serious difficulties, notably by the employment of unqualified women, of inexperienced probationers, and of paupers as nurses. No provision is made in the order for the thorough training or promotion of probationers and assistant nurses, to enable them to rise in the future to the position of superintendents. The effect of this they aver, must be that the general standard of nursing will be lowered instead of raised; while the care of the patients is largely in the hands of insufficiently trained assistants. The disproportion between nurses and patients involves serious neglect of the sick by night. "Also owing to the lack of candidates, the Local Government Board is sometimes obliged to sanction the appointment as superintendents, of nurses who are not trained according to Article III. of the order."

The Association, in conclusion, urged that the serious defects in nursing, lead to frequent and regrettable cases of neglect, and sometimes to tragic consequences; that the absence of a fixed policy, and a uniform standard of efficiency for all infirmaries, as well as of any system of a fixed period of service, leaves the administration to the chance decisions of individual Boards of Guardians, with the result that some infirmaries are well organised, while others are far from being a credit to our civilisation.

### Reports of the Poor Law Inspectors.

In support of their allegations the Association made reference to the reports of the Poor Law Inspectors for 1899-1901 which form, in their view, a strong consensus of opinion on many of the points brought forward; and having expressed the hope that they had shown the necessity for revision of the system in order to secure a better supply of trained nurses, submitted that the Order of August 1897 should be strengthened, and that such other steps be taken as a full consideration of the subject may show to be needful.

### The Signatories.

The statement was signed by Lily Belhaven and Stenton, and F. U. Boyd, M.D., Sibella C. Bonham Carter, W. Chance, M. Fuller, A. C. Gibson (matron of Birmingham Infirmary), Mary S. Hardcastle, F. R. Humphreys, L.R.C.P., Margaret Knutsford, C. S. Montagu of Beaulieu, Margaret Powell, T. Dixon Savill, M.D.London, D.P.H.Cambridge, E. Murray-Smith, M. S. Talbot, Vice-President and Chairman of the Executive Committee, Louisa Twining, Vice-President, H. S. Wantage, J. Wilson, Hon. Treasurer, members of the Executive Committee, and R. V. Gill, Secretary.

### Mr. Long's Answer.

The statement was forwarded to Mr. Long in March, and he had therefore ample time to digest the details. We are glad to learn that in discussing them, he listened to the members of the Association attentively, and that ample time was placed at the disposal of the delegates to set forth their views. The serious dearth of nurses available for the Poor Law service was, it seems, brought forward as the main cause for action, and the Local Government Board were urged to adopt measures for the special training of nurses for workhouse infirmaries. It was suggested that it may be practicable to train nurses in the metropolitan and other poor law infirmaries of adequate size and organisation and bind them to the service, as Government officials, to work for a definite period, in provincial and country workhouse infirmaries as well as in metropolitan. The desirability of a sub-department of the Local Government Board being formed to deal with the whole question of nursing in workhouse infirmaries was also urged by the deputation.

Mr. Long, we are informed, intimated that "he had under consideration the possibility of making alterations in the existing regulations affecting the position of nurses in workhouses and infirmaries." He likewise promised "careful consideration of the suggestions made by the deputation but was unable to hold out much hope that the Local Gevernment Board could themselves undertake the training and placing of nurses." The practical result of this private interview remains, of course, to be seen; but unless, with such eloquent facts before him, the President of the Local Government Board finds it possible, at any rate, to materially improve the condition of the nursing in provincial workhouse infirmaries, he will greatly disappoint the hopes of friends of much needed reform.

### Hints on the Management of Convalescent Homes.

I am the matron of a hospital and convalescent home in the North of England and I have been asked several times how I arrange and plan things here so as to be able to do so much, happily and comfortably, with so small a staff of workers.

### An Ideal Committee.

I think the real secret of our success—I say "our," advisedly—is due to a number of persons who are themselves unconscious of the help they are contributing. To begin with my committee acts as though it trusts me to work well and faithfully without a system of constant inspection and interference such as many have to endure in other institutions. That any member of my committee is liable to walk round and take private friends or other committee members with him, I know, and I should be the last to object to this; but ours is a different system to the irritating one exercised by some, particularly ladies' committees. I am kept informed of our good fortune as well as our less good, and when donations and legacies fall to our share, as fortunately they do from time to time, I am always told of them, so that I shall share the consequent pleasure. In this way we are all made to feel a more than passing interest in the field of our labours.

### The Strictest Economy Observed.

I, too, in my turn try to make those working with and under me interested in our hospital. I have explained clearly in the kitchen our need of the strictest economy in all household commodities, such as a careful use of soap, soda, polish, house-flannels, etc., yet my hospital has many times been denominated "beautifully kept." I have an unusually dependable set of Yorkshire servants who I can assure you summed me up before they accepted me certainly, yet I think the telling point of their character is that having once gained their regard they are true as steel. These servants have realised, as I tried to show them when asking for their help in practising economy in small things, that though unable to give money, they could add to the funds of their hospital by exercising all possible care in the goods committed to their charge. Not only thus does mutual confidence pay in the long run, but it also promotes an *esprit de corps* which all public bodies must know is of infinite value to a community.

### The Co-operation of the Patients.

Each admission day I tell the patients collectively, after reading aloud our few rules, that the committee invite them to eat heartily at every meal, but at the same time mention that they are as emphatically asked not to waste, or allow others to waste, any food whatsoever. To help them to carry out this wish of the governing body, knives are provided with which to cut the slices of bread and butter, should they wish towards the end of a meal to take less than a whole slice. Also at breakfast spare plates are provided for any super-abundance of bacon with which they may have been supplied. This is returned to the kitchen, and thus waste of good food is avoided. Soon after I came here, my attention was called one day to a waste of breakfast bacon, besides several half slices of bread. I myself picked it out of the pig-bucket and had it arranged on a dish nicely and tidily and shown to the patients at the end of a good Sunday dinner. I then asked them to think of a plan by which the home should not lose through their thoughtless extravagance, and one and all agreed to go without meat for breakfast on the following morning. Not once since has there been a repetition of this waste of food, nor did I ever hear one word of grumbling when they breakfasted off bread and butter with golden syrup on the Monday. I hope and believe that the patients realise this is a home for the "people," and that it is to their individual interest to take thought for its welfare, and that it is a point of honour to deal fairly with charity money.

### A Proof of Esprit de Corps.

I was able not long since to put what I call our *esprit de corps* to the test. I was suddenly obliged through illness and accident to lose two servants out of seven. This was a serious matter during our season. My assistant matron and I agreed together to take over all the bread-making and thus relieve the baker for general work. The cook volunteered to let the kitchen-maid do household work, thus taking over kitchen cleaning herself. Even with this arrangement the work could not be done quite comfortably, as I had no one to do the dining-room work which unfortunately belonged to these two sick maids. I did not know how to get over this difficulty, when a knock came to my office door and two smiling young women volunteered to do anything they could to help me, as "they did not like to see me so put about." Here was just what I wanted, a proof of our *esprit de corps*, and a tangible one to boot. These two patients had early meals to themselves and then did the whole of the waiting at each meal for the fifty-six female patients. I was thus easily able to get through six weeks of extra duties for all the servants, with practically no trouble and no expense. Six weeks of extra work in the middle of a season was, I think, a fairly good test of the material I had to work with. Yes, there is good sound grit to be found in a Yorkshire thoroughbred. Some of my readers will say, but why did I not get in outside help instead of trying to work the place in the manner described above? First, it is exceedingly difficult to get servants in a County of mills. Most girls hereabouts prefer to work in mills to being domestic servants. Secondly, I offered a charwoman's help, but my servants said that by the time they had taught her her work they could have done it better and more easily themselves. Moreover, I was glad to have a good reason to show them I was not above doing my own share towards helping when an unusual and unavoidable difficulty arose, and that I knew how to turn to and assist in various household jobs, which do not as a rule fall to the matron's share. I cannot regret my period of inconvenience and worry, for by its means I proved the kindliness of patients, the willingness of servants and the comfort of sister loyal and kind.

### Lectures to Head Sisters.

By E. MARGARET FOX, Matron of Tottenham Hospital.

LECTURE II. (*Concluded from page 263.*)

If there is one point more than another in which the good or bad management of a ward is apparent, it is in the manner in which the meals are served, and hospital methods of serving food often leave very much to be desired. Patients' breakfasts, for instance, are sometimes given in rather a hap-hazard way, owing to the night nurse being in a hurry to get through the morning work, and when they are roused suddenly from sleep, and expected to take what possibly is very unceremoniously set before them, or are kept waiting for one thing and another, it is not surprising if sometimes they refuse it altogether. Bread and butter for the six o'clock breakfast should be cut early, and covered with a clean cloth until wanted. Eggs should be collected, saucepan and kettle filled, plates and mugs ready on a tray long before you need them, so that no time may be lost when the time comes to give them round.

If the patients' breakfast is at 6 o'clock, and their dinner at 12.30, then it is important they should have their lunch quite early, about 9.30. If you allow your probationer to give it between 10 and 11, you will find very bad appetites by dinner-time. Of course, you will not give them more than milk, hot or cold, beef tea, and a piece of bread and butter or a biscuit, unless they are on special diet. It is a mistake to make arrowroot, cocoa, etc., for patients' lunches, as it takes too long in the morning, and can be better done at supper-time, when the work of the day is behind you. Dinner, being the principal meal of the day, should not be hurried over, as it is quite an event to the convalescents at any rate, and they should have time to enjoy it. See that your probationers go round beforehand with bibs, cloths, and bed-tables, and that knives, forks, and spoons are given out as required, which saves time when it comes to the actual distribution of the food.

Always serve out the dinner yourself, unless something unusual prevents you, and then you will be sure that each one gets the right diet. Be particular that salt, bread, and something to drink is given at the same time as the plate of food, and that it is hot and neatly served. Have the plates taken first to those who can feed themselves, but in the case of children, it is best to get all the plates ready in the ward kitchen and then to carry them round to each child, so that the smaller ones may not be crying ever so long because they see an older one having its dinner while they have to wait to be fed.

Ward teas are best given between 3.30 and 4, the same method being followed as in preparing breakfast, and suppers are usually served about 7. Then is the time to humour the patients' fancies a little, and many a one with a poor appetite who will turn with loathing from an egg beaten up in milk, can be coaxed by a little boiled custard or an egg cunningly concealed in a cup of arrowroot or coffee Also, if a patient has been kept without his dinner in view of a possible operation which did not take place, you will earn his everlasting gratitude if you have had his dinner saved for him and give it to him at supper time just as he was feeling miserable at having missed the chief meal of the day.

The points I would like to impress upon you in the serving of meals is to serve them punctually, quickly, and appetisingly, and afterwards to have them cleared out of the ward, and the washing up done as soon as possible. No one cares to lie and look at empty mugs and plates, and if they are left about after meals are over they give a very untidy appearance to beds and lockers. See that trays are used for carrying mugs, etc., in and out of the ward; that crumbs are brushed out, and beds tidied after meals; and that the food is not wasted, either by nurses or patients.

In hospitals where the breakfast is given by the night nurse, mugs and plates should all be washed and put away before the day staff comes on duty, the beds made, and those patients washed who are able to wash themselves.

The head sister should, as soon as she has heard the night report,, go round her ward, speaking to each patient, and noticing if everythiug is left as it should be by the night nurse; if the ward and various offices are tidy, the fire bright, hot bottles refilled, poultices and fomentations renewed, four-hourly temperatures charted, and children clean and dry.

She should see that everyone gets to work at once, without wasting any time. Twenty minutes lost in idle gossip at the beginning of the day means an hour at the other end. You can never catch it up. It is so easy to get into a dawdling way of doing things, and the minutes slip away fast in the early morning. It is necessary to work with one eye on the clock all the time if the time-table you have arranged for yourselves is to be adhered to. By 10 o'clock on an average day and with sufficient help, the ward should be in perfect order. Sweeping and dusting should be finished, flowers arranged, fires bright, the morning temperatures charted, the patients all washed, bedpans distributed, the beds tidied, and lunches given round; but this cannot be done unless every worker puts her best foot foremost.

By 12.30 lavatories and bathrooms should be cleaned, the ward and kitchen tidy, and the patients ready for their dinner. If the doctor has paid a reasonably early visit, then dressings should all be done and cleared away, instruments boiled and dried, lotion bowls and dishes washed and in their places. The patients' dinners should all be finished by one, and then between one and two bedpans should be given, beds tidied, fires made up and hearth swept, convalescent patients dressed and their beds made, and the ward lightly swept and dusted.

During the afternoon, of course, there may be operations, new admissions or doctor's visits to occupy your time, but if none of these things occur, and the ward is pretty quiet, splints should be padded and ward linen mended. By 5 o'clock the tea things should be washed up, and those patients who are allowed out of bed for a short time should be got up and their beds made. Between 5 and 7 is the time for sponging typhoids, washing backs, giving vapour baths, etc., taking evening temperatures and preparing suppers, which should be cleared out again by 7.30. If you are a good manager you will arrange to have no one off duty during those two hours, when there is always a great deal of work to be done. Half-past seven is a good time for ward prayers, as if they are left till 8, you may be tempted to omit them altogether, some of the patients having gone to sleep, and you perhaps are feeling tired and worried because the work is not finished.

At 8 o'clock turn down the lights, and allow no more talking among the patients, and do not make the hour between 8 and 9 an occasion for exchanging confidences in the ward kitchen. Write the night report in good time, and by 9, see that everything is left in order for the night nurse. Leave ready for her use all that you require her to leave ready for yours, and strive for order, method, punctuality, and attention to details, in every particular.

Good ward management, like everything else worth knowing, is not mastered in a day; but if you are in earnest you will learn by your mistakes, by your failures more than by your successes, by your difficulties and the way you mee tthem, and above all, by attention to the so-called "little things." It has been said that "a little thing is a little thing, but faithfulness in little things is a very great thing."

### The Sterilization and Storage of Milk.

By HELEN TODD, Matron of the National Sanatorium, Bournemouth.

It is said that the English nation is almost the only one which habitually consumes uncooked milk. Recently, however, raw milk has been demonstrated to be by no means the harmless fluid it was generally supposed, but on the contrary, it has been frequently proved the chief agent in the spread of various infectious diseases, such as diphtheria and enteric fever, the germs of which can live and thrive in it. It is also now believed to be a common means of conveying the tubercle bacillus from infected cows to the human race. Whole epidemics of scarlet fever have had their origin traced to a local dairy, and children fed on milk from cows suffering from "foot and mouth" disease have been known to develop a form of stomatitis.

The public danger in this respect is fully realised by many foreign governments, and in some countries, as in Denmark, special legislative measures have been passed dealing with and controlling the sterilisation of milk.

Sterilisation can only be performed satisfactorily by the application of heat. Various drugs such as boric acid, have been tried but with disastrous results, especially when the milk so treated had been administered to the sick or young children. The rough and ready method of boiling milk for a few seconds can be relied upon to render it harmless, but, unfortunately, it becomes at the same time less nourishing and somewhat unpalatable.

Many persons object strongly to the flavour of boiled milk, and in cases where patients are on an exclusively milk diet the difficulty to the nurse is a very real one, for hardly anyone in health would drink tea or milk and soda if prepared with milk that had been boiled, and much less would they touch it when ill. A little trouble will, however, overcome this difficulty and render the milk both harmless and free from the unpleasant taste caused by actually boiling it. It has been proved that a temperature of 190° Fahr. maintained for 10 minutes is sufficient to destroy most bacteria, including the tubercle bacillus. Milk so treated does not alter appreciably in flavour nor does any skim form on the surface if the operation be properly carried out.

In hospitals and other lay institutions where a large quantity of milk is consumed it is as a rule sterilised by means of a special contrivance; where steam is used for cooking purposes a jet may be caused to pass through the milk or it may be heated in a large reservoir, usually of copper tinned inside. The most practical of these, of which there are many different patents, consists of a large water chamber heated by gas which generates steam enough to surround the milk contained in an inner vessel and so bring it to the required temperature.

In private work, the nurse will, of course, rarely find such an elaborate contrivance. The most convenient plan of "sterilising" milk is to place it in a covered jar, or tightly-corked bottle, inside a saucepan of boiling water; the jar or bottle should be provided with a thermometer coming through the cork or lid, and the water in the saucepan must be kept boiling until the thermometer registers 190° Fahr. It should be kept at this heat for 10 minutes, and then the milk must be quickly cooled by substituting cold water for the surrounding medium. If this be not done, and the milk is allowed to cool slowly, the cream will "separate," either forming an almost solid crust or floating on the surface as a greasy oily fluid, rendering the milk most unpalatable. The jar or bottle containing the milk must be hermetically sealed before undergoing the heating process, and on no account opened until the milk is required for use, otherwise fresh germs may find their way into the milk from the atmosphere. Milk so sterilised and sealed will keep some days without showing signs of fermentation.

It is very necessary to choose with some care the place where the daily milk supply is to be stored, for not only is it liable to contamination from disease-bearing dust, but it has also the peculiar property of absorbing sewer and other gases and vapours which may be extremely dangerous to the health of the consumer. Boiling milk so contaminated does not render it innocuous and so fit for human food.

The common phenomenon of sour milk is due to the fermentive changes caused by certain bacteria which convert the sugar in milk into lactic acid, which in its turn coagulates the casein; these changes occur much more rapidly under certain atmospheric conditions, if the milk be kept warm, or if it be from the udder of an unhealthy cow. The bacteria find their way into the milk from the atmosphere, but are fortunately easily destroyed by heat.

It should not be necessary to remind nurses how important it is to see that all vessels which are intended to contain milk are kept scrupulously clean, and really boiling water, with plenty of common soda dissolved in it, used for their cleansing. It has even been said that cases of enteric fever have been traced to the washing of milk cans with infected water.

In district work especially it is well for the nurse to bear in mind that very frequently indigestion, diarrhœa, and other infantile maladies have their source in the inefficient cleansing of bottles, teats, or those horrible lengths of indiarubber tubing still used by the lazy and ignorant in the old-fashioned type of baby's bottle.

The boat-shaped bottle, open at both ends, (one being fitted with a cork and the other with a teat without any tubing connection), can alone be recommended for a baby, as it is the only bottle that can be thoroughly cleaned. Two bottles and teats should always be in readiness and used alternately. After a meal the bottle must be carefully cleansed with boiling water and soda, and it should be kept in a basin of clean cold water until it is next required.

### Presentation and Garden Party at Llangarran.

By kind permission of Mrs. Williams, President of the Fisherton Auger Nursing Association, a garden party and sale of fruit and vegetables was recently held in the grounds of Llangarran. The function was the first of its kind held under the auspices of the Association, and with the proceeds it is proposed to provide nourishment for the sick people in the district. After short addresses by the Rev. E. N. Thwaites and Mrs. Williams, the latter declared the sale open. Later in the day Mrs. Williams presented a certificate of the Royal Humane Society to a lad who had distinguished himself in saving life. The treasurer, Mrs. R. C. Harding, explained the circumstances. Some children were playing on the bank of the river near Black Well when one of them fell into the deep water. Albert Dyer, a boy of 14, pluckily went to the rescue, dived in, and brought out the child, thoroughly exhausting himself in the act. At this stage George Humphries, a lad who had been reading directions for restoring animation to the apparently drowned, promptly put his knowledge to the test, and completed Dyer's plucky efforts by restoring the child to life. Dyer on a previous occasion had effected a rescue. These facts came to the knowledge of Nurse Botham, who communicated with the Royal Humane Society, and obtained their certificate and gift of 10s. as an additional reward. The meritorious services of Humphries were not overlooked. The presentation of the awards proved an interesting feature of the proceedings, which resulted in a profit of £11 10s. 8d.

### A Book and its Story.

### THE COUNTRY OF THE HOUR.*

SOUTH AFRICA of to-day is a country whose very name is one synonymous with unrest, tumult, disorder, and upheaval; the upheaval of the old tyrannical policy of obstruction and oppression giving place to one of enlightenment and freedom, under the beneficent direction of the British Crown. "South Africa a Century Ago," as revealed by the letters of Lady Anne Barnard, might have been written at the close, instead of at the opening of the nineteenth century, from the curious resemblance borne between many of the difficulties of the moment, and those of the present day. "The same problems presented themselves for solution; the same difficulties arose, and perhaps the same mistakes were committed on either side."

A peculiar interest attaches to the Letters, as they cover the correspondence of two distinguished personages of the period, viz., the wife of the first Secretary of Cape Colony, and the first Viscount Melville, Secretary of State for War under Pitt. Lord Melville was the one statesman of the period who realised the importance of the Cape to us, not only on account of its valuable internal resources, but also as being a station of first importance on the line of our route to India. "No man in Great Britain," we are told, "paid more attention to the affairs of the Cape or had more intimate acquaintance with every subject relative to the Colony." Lady Anne and Lord Melville were old friends, and in appointing her husband, Mr. Barnard, to the post of Secretary to the Colony, Lord Melville did so with confidence in the many social and intellectual gifts which made her admirably fitted for the position of first lady of the colony, as Lady Macartney, the wife of the Governor, was not accompanying her husband. Lady Anne was charged to conciliate the Dutch in every way and to write freely about everything that occurred. That she fulfilled her mission with a generous impartiality is shown by her letters. They prove keenness of perception, a liberal mind, and through all runs the magnetic thread of a sympathetic personality directing and controlling the important part in affairs which she was called upon to fulfil. The correspondence extends through the administration of the two first English Governors—Lord Macartney and Sir George Young. It shows throughout much shrewd wisdom in suggestions for the conciliation of the Dutch and the government of the colony, more particularly with regard to the treatment of the natives. For over one hundred years these letters have slumbered among the archives of Melville Castle, and the public is indebted to the present Lord Melville for their opportune appearance. In the waning light of the present struggle they have an added interest. Not the least admirable portion of the book is the brief memoir written by the compiler (Mr. W. H. Wilkins, M.A., F.S.A.), with a short succeeding summary of Cape history from its discovery, in 1486, to the first appearance of the English flag in Table Bay in 1591. It was not until the decline of the Portuguese power that the Dutch established themselves in the East, and the Cape was not colonised by them until 1652, by the order of the Dutch East India Company, the settlement taking the form of a military outpost to start with. These early settlers were made up of a motley crowd of Flemings, Germans, Poles, and Portuguese of a low class. Later, in 1686, on the revocation of the Edict of Nantes, an important addition was made to the little colony by the addition of a number of Huguenot refugees. This immigration is still to be traced in names still existing in South Africa, such as Joubert and Du Plessis, both old Huguenot names. For the next century the country was under the misrule of the Dutch East India Company. They seized the territory of the Hottentots, and reduced them to the position of serfs; they introduced Malay and negro slaves into the Colony; and harassed the settlers with needless and harassing taxes. They specified to the farmers the crops they were to grow. The appeals of the settlers to the Dutch East India Company met with no result until their several attempts to throw off its rule ended in the rebellion of 1795. There were in Holland two parties with differing sympathies towards the insurgents—the "Orange party," which favoured the Stadtholders, the Prince of Orange, and the alliance with England, and the "Patriot" party which held Republican principles and sympathised with the French. At this juncture an expedition was despatched from England under Admiral Elphinstone and General Craig. After a short engagement in June, 1795, the Dutch capitulated to the English, and the English took posssession of the Cape of Good Hope.

It is from this point in the history of Cape affairs that Lady Anne Barnard's letters date. A comment appears in her first letter which bears on the social aspect. An English officer was on board who had married a Dutch wife. This lady refused to be conciliated, and was finding much fault with the Government for sending to the Cape "a parcel of people who cannot please the Dutch or be likely to adopt their manners." "I hope," adds Lady Anne, "that her manners are not a specimen, and that her calculations will not prove good, as the Dutch are said to be eager to be well with the English . . . . at any rate, Mr. Barnard and I mean to do all in our power to conciliate them as much as we can, and meet the habits and customs of the place half way." One of their first steps was to make a journey to the interior. The lively and graphic description of their adventures by the way take up the greater part of the letters. Space does not allow of many further references to the contents of this most entertaining journal; but one of a visit to the Moravian Mission to the Hottentots is instructive. "They asked us to step in and see the church . . . presently, at sunset, the bell rang for prayers . . . I doubt whether I should have entered St. Peter's at Rome, with the triple crown, with a more devout impression of the Deity and His presence than I felt in this little church, where the simple disciples of Christianity, dressed in skins of animals, knew no pride and hypocrisy. I felt as if I was creeping back seventeen hundred years and heard from the lips of the inspired Evangelists the simple sacred words of wisdom and purity. They then sang the 23rd Psalm . . . in a tone so sweet and loud, so chaste and true that it was impossible to hear it without being surprised."

As Lady Anne Lindsay, Lady Barnard was one of the most interesting and picturesque figures in the Society of the period, and she, and her beautiful widowed sister, Lady Margaret Fordyce, were known as the "Lindsay Sisters," whose home was a social centre for the world of fashion and letters. Lady Anne was a great story-teller. Once, during an untimely pause between the courses at a dinner party of distinguished guests an old servant came up to her, and whispered, "You must tell another story, my lady, the next course will not be up for five minutes." To her many personal charms she joined also a pretty gift for verse, and the time-honoured ballad "Auld Robin Gray" owes its origin to her facile pen, and to a passing mood of melancholy. Her sister, Lady Margaret, had just married. Through Lady Anne's ears was running the haunting melody of an old song sung to her as a child. It was one for which she had a particular affection. To solace her loneliness she composed the plaintive words to fit the tune, little dreaming of the wide popularity they would attain. With her little sister as audience she recited the poem, not as yet completed. The heroine had lost her lover, her father had broken his arm, her mother was ill, and old Robin Gray appears in the extremity. The authoress, feeling her own bereavement was great, wished perhaps that her heroine should have a balance of misfortunes to outweigh her own. No other calamity suggested itself until, addressing her sister, she said, "I wish to load her with a fifth sorrow, poor thing!" "Help me to one, I pray." "Steal the cow, sister Anne," said the little Elizabeth. The cow was at once *lifted* by me, and song completed. Here we must leave Lady Anne and trust our readers may avail themselves of the opportunity given by her letters, of meeting her on her own ground.

* "South Africa a Century Ago." 1797-1801. By the Lady Anne Barnard. (Smith, Elder & Co. 1 vol. Price 7s. 6d.)

### Echoes from the Outside World.

### AN OPEN LETTER TO A HOSPITAL NURSE.

THE Queen has done more for British industries by her letter to Lady Amherst of Hackney than all the "Fair Traders" put together. In expressing the "earnest hope" that all ladies who are to be present at the Coronation will employ for their dresses, "as much as possible," materials made by British manufacturers and embroidered by British work-women, her Majesty has, it is true, but followed her own example as Princess of Wales. The Coronation of the King and Queen will, however, be such a great event that the question "What shall we wear?" must be of profound importance to all the ladies who are to enjoy the privilege of participating in it. After the gracious intimation of the Queen, it may at any rate be assumed that the national industries, in the welfare of which we are all more or less interested, will be requisitioned for the supply of a large proportion of their garments.

I SEE that the directorate of the Kurhaus at Homburg have made King Edward an appropriate little present. It is a special tumbler from which he can drink his morning glass of water from the Elizabeth Spring. It is made of thin fine glass, and a scale is engraved upon it the same as upon an ordinary medical glass. There is a handle and the Arms of England are emblazoned upon the front. The same kind attention has been paid to the Duke of Cambridge, and the gifts have been offered in consequence of the new rule, about which there has been much discussion, namely, that every visitor must possess a glass of his, or her own, to be used exclusively by the owner. It is rather difficult to understand why there should have been any opposition to such a sensible regulation in these enlightened times, when even the most ignorant are aware that disease is caused by microbes, which are easily multiplied and spread. I remember well, when I was at Carlsbad some years back, watching the people sipping their matitutinal beverage, being struck with wonderment that comparatively so few regularly brought their own glass or mug. Personally, I am sure that I should have felt a qualm of disgust and fear had I been obliged to drink after some of the poor disease-stricken mortals whom I frequently saw sipping the water, and yet it never seemed to have entered the head of many who went seeking to cure one ailment that they might possibly at the same time be contracting a second. I should think it will not be long before the regulation at Homburg becomes the rule at every watering place.

SINCE last I wrote to you we have moved on to Weymouth. On our way we stopped an hour or two at Dorchester, principally because I have a special craze about which I am often teased—I like to become acquainted with the capitals of our counties whenever I can. I think the idea dates back to the time when the counties of England and their capitals was almost a daily lesson in the schoolroom. In its way Dorchester is rather a charming place, and reminds me of a foreign town more than a prosaic English one, for it has avenues of trees everywhere. In some of the main thoroughfares the trees are still small, so that in the future the effect will be even better than it is now, but in others they are already large enough to meet overhead, and these "Walks," as they are called, with their seats dotted about, are a most pleasant feature. The West Walk boasts a piece of the old Roman wall, and the South Walk has some lovely chestnut trees. There are also the Borough Gardens with flower beds, a band-stand, and lawns where tennis and croquet may be played at a trifling charge. The house of Mr. Thomas Hardy, the novelist, close to the old Toll Gate, is pointed out with pride to visitors.

WEYMOUTH itself is a very important place, and the bay singularly beautiful. It is said to closely resemble the Bay of Naples, and has an enormous sweep, so that of course the sea promenade is unusally long. One peculiarity of the place is that it is almost impossible to be very far from the sea wherever you may be located, even the South-Western railway station being only four hundred yards from the front. This is because there is so little depth to the town, as a tidal backwater, about two miles long and nearly half a mile wide confines the western portion, and the streets are naturally stopped by the water before they can grow to much length. This backwater expands after it passes the bridge into a large lake three or four miles long, called Radipole Lake, where there are dozens of swans. Along the Esplanade there are some large houses, which with such a splendid view and handsome rooms can command good prices, but it is possible to get accommodation of almost any kind desired, and some of the houses in the quieter streets are by no means expensive. For those who love bathing, except at very low tide, Weymouth is quite a paradise. Naturally, it is seldom as rough in the bay as it would be outside, and I have never stayed in a town where more is done for the convenience of the visitors. There are plenty of roomy clean machines; there are four bathing saloons, two for ladies and two for gentlemen, where the charge is only 2d. a person. At Greenhill there is a bathing shed for men which they may use free, and for a nominal sum possessors of tents may pitch them on the sand. People who are good sailors have a splendid time at Weymouth, for there are such a large number of excursions by sea, amongst others to Jersey and Guernsey, Swanage, Lyme Regis, Lulworth Cove, and Bournemouth. Those who prefer to stay quietly at home will find ample pleasure in drinking in the fresh pure sea breezes, and in the walks around, which are sure to include one to see the White Horse, the figure of an equestrian standing out on the hill-side, made by cutting away the grass and earth into this particular shape, and so exposing the chalky subsoil beneath.

I WONDER what the general opinion of my readers is upon the subject of the fashionable craze for allowing children to relinquish their shoes and stockings in favour of sandals. For some few years past up-to-date mothers have kept their babies without covering for their feet, allowing the mites to kick about unfettered in their perambulators, and even when larger to run about the house in the same condition. But the present demand for sandals in towns seems due to fashion rather than to hygiene. No one knows how it originated, or who it was who led the van, but the fact remains that 1,500 pairs a day has been the turnout of one Leicester manufacturing firm alone during their busiest time. Physicians are said to be in favour of the innovation, because it allows the foot of growing children to expand naturally, but then they probably assume that sandals once adopted the wearer will no more return to the ordinary stocking or sock boot or shoe. I believe that this idea is wrong. With most mothers it has been the novelty which has been the attraction, and after a short time a great many will find that little girls attired for church or party going, with leghorn hats smothered in white feathers, clad in silk and lace frocks, look a trifle incongruous with bare legs coming out beneath; that the boys frequently return home after a country walk in sandals with cut big toes and scratched feet; and that instead of walking with a free springy step, as they do either barefooted or in well-fitting shoes, the children in sandals are very apt to shuffle as they walk because the flat piece of leather feels loose and apt to slip off. Anyhow in London it will only be in the summer months that the absence of boots will be popular.

### Everybody's Opinion.

[Correspondence on all subjects is invited, but we cannot in any way be responsible for the opinions expressed by our correspondents. No communication can be entertained if the name and address of the correspondent is not given, as a guarantee of good faith but not necessarily for publication. or unless one side of the paper only is written on.]

### A BELGIAN NURSING SISTER ON THE BRITISH SOLDIER.

"Mme. Alice Bron," a nursing sister, writes from No. 10 Stationary Hospital, Naauwpoort, Cape Colony: For patience and endurance it is impossible to surpass the English soldier and he amply repays in gratitude and respectful affection those who give to him the best of their knowledge, their strength, and their devotion. Personally, and much more than an English sister, I am able to appreciate the tact and the *savoir vivre* of Tommy Atkins, for, speaking English very little as I do, it would be the easiest thing in the world for me to become a subject of amusement. Yet, on the contrary, I have never perceived even the shadow of a smile, nor the least expression of amusement pass over the faces which have always been so friendly to me. Last year at Jacobsdaal, when it fell to my lot to take care of such a large number of English wounded combatants, I was struck by the same thing; but then there was a constant change in the suffering patients, fresh ones taking the place of the older very frequently. As I was the only nursing sister, assisted by two R.A.M.C. orderlies, I fancied that it was the comfort of feeling the gentler touch of a woman's hand which made them for the time so pleasant and courageous, and that were I to have seen more of them, I should have found cause to modify my opinion. I was therefore full of curiosity and anxiety—fearful that my good opinion should be dissipated—when I took service as a regular army nursing sister in South Africa. But I found that mine was to be the pleasure of finding my first impression confirmed, enlarged, and deepened, and I have now been here for some months. The English soldier is just exactly what I had judged him to be last year at Jacobsdaal, and I shall always regard him as a dear and precious friend who causes one to forget fatigue, and who turns even the most disagreeable services into a pleasure. Both at Capetown working under the surgeon, here attending the enteric, the dysenteric, the pneumonic cases, sick, dying or convalescent, he is always the ideal patient. The orderlies, too, are very good to the foreign sister; my companions help me in every way they can, so that, more than ever, I am devoted to my hard and sweet vocation, and yet more happy if I can avenge by such means as lie in my power all the base calumnies and the odious lies which are circulated about England by a press, a portion of which has sold itself, whilst the other portion is both blind and deaf.

### PRIVATE NURSES' FEES.

A "Private Nurse" writes: Both "A. B." and "A Sympathiser" put all the blame for the "dreadful expense" of a good nurse on nurses' homes, and "A Sympathiser" goes further and blames them for the faults and failings of nurses. Now, is this just? To take the last indictment first, "A Sympathiser" says, "Doctors would have better nurses, inasmuch as the nurses would have a greater inducement to work well if on their own account." I may have misquoted these words but the sense is correct. Surely this is a mistake. A nurse who cannot work as well and as conscientiously for the institution to which she is attached as for herself is not a good nurse. Would a nurse ever be respected who said, "In this case I am only a member of a home; perhaps the doctor does not even know my name, therefore I need not do my best." But to go back to the first statement of "A Sympathiser" and the chief grievance of "A B.," *i.e.*, that no nurse can be procured under £2 2s. weekly. This is a mistake. In London it may be the case, but in the provinces the usual charge is from £1 5s. to £2 2s. for the highest fee. "A Sympathiser" also states that after infectious cases the nurses are not sent into quarantine. But the usual rule is for nurses to be sent for a week or more, as the case may be, to rooms set apart for that purpose. Then she goes on to say, "No benefit accrues to the (salaried) nurses." On this point I do not speak from "what I hear on all hands," but from personal knowledge. The work is hard, but in all respectable homes—there are some inferior ones I grant—the food is good and plentiful, and the nurses are entirely free from all the worries of providing and housekeeping generally. Of course the housekeeper advocated in "A Sympathiser's" letter might manage well; but, on the other hand, she might not. In a home the superintendent has all these things to see to, besides being responsible for the business of such an establishment, which must necessarily be considerable. Would the plan of taking a house between six or eight nurses be more comfortable, or even more profitable for them? First, they must get a good housekeeper, and she would certainly need help, as well as a little domestic probably. Thus, the wages of two people and their board have to be taken into consideration; and it must also be remembered that the housekeeper's salary will be high, because she would need to be an intelligent, well-educated, woman, able to deal with the daily business of such a club. Of course, all going well, even then expenses, and rent and rates might still leave a margin greater than £30 per annum clear. But the great difficulty is having no one to fall back on. A nurse gives offence to a patient, perhaps through no fault of her own, and they request her to go. Who will stand up for her? The doctor may, but it is unlikely. Certainly nurses would gain independence under such a system but independence is not the best thing in all cases; it is as well to have someone to depend on. I see in your "Notes and Queries" that a nurse asks how she can get her unpaid fees—and the answer is "go to a solicitor." If that nurse had belonged to a home, she would have only had to appeal to the home for redress, and as a salaried nurse it would of course have made no difference whatever to her. And, lastly, in case of sickness, what a dead loss it is to nurses on their own account, whereas the salaried nurse loses nothing. Surely, when one considers all these things fairly, the case against "Nursing Homes" falls to the ground and it must be owned that the nurse does receive "personal benefit" by belonging to one.

### NURSES WHO SHOULD BE WEEDED OUT OF THE PROFESSION.

"An Admirer of Nurses" writes: I notice in one of your recent issues a letter from "The Matron" of a nurses' home in Preston. She asks, "Can anything be done to prevent nurses remaining in the profession when they have disgraced both their uniform and sex by misconducting themselves?" I say there can, and in this way: when a nurse applies for a place in a home let the lady superintendent take her hospital certificates and keep them until she is leaving; then, if she is sent in from any private case for misconducting herself, write across these certificates, in red ink "discharged for misconduct." I think that this could be easily brought about. I have had a great deal to do with nurses in one way or another, and could give several instances where nurses have sacrificed themselves over and over again for the sake of the patient. For example, when I was seriously ill a few months back I had a private nurse who was most attentive and could not have been more so even if I had been her own brother. I am now in a consumption hospital and have got another such as she—nothing seems to be a trouble to her. Another nurse I know of was nursing a wee boy who was dangerously ill. She used to stand, not sit, hour after hour, through the night, fanning him. The father saw this with his own eyes unknown to the nurse. Still another nurse I know of went out to the front; there she used to sit reading to her poor wounded "Tommies," write letters for them, and saying comforting words to them now and again. She caught enteric fever, and was invalided home; got better, and then volunteered for China. The Government doctors would not let her go, but made her matron of one of their hospital ships, bringing the wounded home. In each of these cases there has been the "pure self-sacrifice," which is characteristic of all good nurses. They very seldom think of self—it is always, "What can I do for the comfort of my patient or patients?" On the other side, I know of a nurse who was private nursing and had a bad case. She asked the mother of the patient to stop out of the sick room, as the patient was always much better when she did. The mother refused, and found out afterwards that it wasn't for the sake of the patient she wanted the room to herself, but in order that she could take the wine, etc., that was set aside for the patient. The end of this nurse was death through drinking. Of course there are black sheep amongst every flock, and those of the public who put down nurses as being a bad lot because they know of one or two misbehaving themselves, haven't had much to do with them, else they wouldn't say such things. I think we should all be thankful that we have such "ministering angels" to fall back upon.

MALARIA AND MOSQUITOES.

"Mrs. Edith Billington" writes from the Congo: As soon as The Hospital, *Climate*, and *Le Mouvement Géographique* arrive I look out for all I can find on "Mosquitoes," "Malaria," and the "Tropics," and have just been reading an article on "Malaria" in your issue of March 16th. Please let me say that mosquitoes most certainly do bite goats, on the Congo at least, for every night when the mosquitoes are particularly troublesome we hear our poor goats kicking about badly, and sometimes they kick so much that their noise in their wooden house keeps us awake. I must try, when I get an opportunity, to find out whether the right kind of mosquito gets at them, and whether it leaves any traces in their blood. If it does, I suppose the next thing will be to provide them with mosquito-proof houses in self-defence, as we are so dependent on them for milk, and sometimes for meat. Since March I have had a little rat terrier, and she feels the mosquitoes badly too; indeed, while on our little mission-steamer she would creep away at night, after killing as many mosquitoes as she could, into a locker under one of the beds. I fancy we shall hear by-and-by that mosquitoes, flies, ants, cockroaches, or some other insects, possibly bees, are responsible for spreading more troubles than we had imagined. How people can eat native honey in this country I don't know, for African bees seem to feed on all the kinds of filth they can find! And yet the natives eat the honey, black as it is, and wonder that we will not buy it. All these insects seem to feed on rubbish heaps, etc., in the natives' quarters, and then run all over people's food if they have a chance. Then as to water, some stations depend largely on rainwater tanks for drinking water, and I have known these to be quite open. Would it not be better to close them altogether, or to put on a lid that could be removed for weekly inspection? It is certain that mosquitoes will breed wherever one leaves them a little water undisturbed; I usually empty ewers, bottles, etc., if leaving the house for a day or two, but have seen them growing in a flower-vase that was overlooked! *Re* "Quinine Pills," in your issue of March 30, I think all Congo missionaries have given up using them in fevers for many years. Tabloids of bi-sulphate of quinine are usually more reliable, but latterly we have used McKesson and Robbins' capsuled pills.

A NURSE'S SUGGESTION TO PUBLIC BODIES.

"A District Nurse" writes:—While reading "A Nurse's Suggestion to Public Bodies" in your columns, I was struck by the practical way "A. M. A." dealt with the subject. However, it is by no means a new idea. The same subject of providing temporary isolation hospitals for rural districts, suggested itself to me while nursing smallpox in a very small cottage. No one, excepting those who have nursed in small cottages, knows the great inconveniences and disadvantages to be met with there. For instance, the nurse when off duty often has to sleep in the kitchen, where the patient's meals are prepared, and cooking and cleaning to be done Often there are only two bedrooms in a cottage—these divided by a thin partition of match-wood. Supposing a nurse preferred sleeping in one of these rooms, so as to be near her patient, the husband has, perforce, to sleep downstairs. This is still more inconvenient, as in preparing food and heating water, it is necessary to use the kitchen, unless a lamp or stove is kept in the bedroom—and this is most objectionable in hot weather. I can fully sympathise with "A. M. A." as to the sleeping arrangements, having known what it is to try to sleep on an old wooden couch, pestered with fleas, which I could not get rid of after an hour or more scrubbing with carbolic acid; then after a night, still weary, I was up about 5 A.M. attending the patient. Such discomforts might be avoided if, say, a moderately sized house, or iron building, was taken by the Local Government Board during an epidemic and used as a temporary hospital. In many rural places I know there is no isolation hospital, but surely an empty house with good sanitary arrangements would be vastly preferable to nursing these poor people in their own homes and amongst their own people. Often relations, willing to render assistance, do much harm by giving their loved sick ones things which they ought not to have. For instance, on one occasion I found a bag of common sweets under my patient's pillow, which the husband surreptitiously had given her, though she was so ill that it was with great difficulty she could swallow any solids, with a temperature of 104° and higher at times. Then again, when the patient dies, how sad it is to see the body brought down to the kitchen, or, worse still, left in the same room shared by the living, and this for two or three days previous to burial. Surely something might be done to avoid such risks of infection and to lessen the nurse's arduous work.

### Appointments.

Accident Hospital, Mountain Ash, North Wales.—Miss Mackay has been appointed matron of the above hospital. She previously held the post of assistant matron at the Chelsea Infirmary.

Grantham Hospital.—Miss Evelyn Hastie has been appointed night charge nurse. She was trained at the Westminster Hospital, and worked there for five years. Subsequently she was nurse at St. Luke's Hostel, and charge nurse at the Thames Ditton Cottage Hospital.

Leek Memorial Cottage Hospital.—Miss Sara Denniston has been appointed staff nurse. She was trained at Drumcondra Hospital, Dublin.

Private Hospital, West Hartlepool.—Miss Tillie Fitzgerald has been appointed nurse in charge. She was trained at the Royal Victoria Hospital, Belfast, and was subsequently charge nurse of the casualty wards in the same institution, and charge nurse in the surgical wards of the County Infirmary, Downpatrick.

Royal Albert Hospital, Devonport.—Miss Gertrude Savage has been appointed sister. She was trained at Gravesend General Hospital, and has since been charge nurse at the Hertford British Hospital, Paris, staff nurse at Poplar Hospital, and staff nurse at Monkwearmouth Hospital, Sunderland.

Women's Hospital, Sparkhill, Birmingham.—Miss Ethel Evans has been appointed staff nurse. She was trained at Burton-on-Trent Infirmary, where she was afterwards staff nurse. She has since been engaged in private nursing.

Worcester General Infirmary.—Miss Lucy Evans has been appointed sister at this institution. She was trained at the London Hospital and has since worked as private nurse and on the staff of the Worcester Infirmary.

### TRAVEL NOTES AND QUERIES.

Sea Voyage (Nurse).—I wish you had asked me earlier, I am now away from London, and unable to obtain information; but this much I can tell you. There is no trip that I know of that will last nearly a fortnight and no more; in all cases, I think, you would be compelled to land, and it would be expensive. There may be such trips, though I do not know of them. Write, and ask Messrs. Gaze and Sons, Tourist Agents, Piccadilly, W., and use my name. The next best thing I can suggest is to go to Knokke, in Belgium; you will find all particulars in Nursing Mirror of September 1st, 1900.

### For Reading to the Sick.

A THANKFUL HEART.

WHEREFORE I cry, and cry again;
And in no quiet canst Thou be
Till I a thankful heart obtain
Of Thee.

Not thankful when it pleaseth me;
As if Thy blessings had spare days;
But such a heart whose pulse may be
Thy praise!
*Herbert.*

The soul, to God's heart moving on,
Owns but the Infinite for home;
Whatever with the past has gone,
The best is always yet to come.

'Twill not be growing old, to feel
The spirit, like a child, led on
By unseen presences, that steal
For earth the light of heavenly dawn.

To wait—to suffer—or to do;
Each key unlocks its own deep bliss;
For every grief a comfort new—
A mine for gems the heart may miss.

. . . . . . . .

Heaven's inmost sunshine earth has warmed;
Heaven's peace floods each dark mystery;
And all the present glows, transformed,
In the fair light of what shall be.
*Lucy Larcom.*

IT is a tremendous moment when first one is called upon to join the great army of those who suffer. That vast world of love and pain opens suddenly to admit us one by one within its fortress. We are afraid to enter into the land; yet you will, I know, feel how high is the call. It is as a trumpet speaking to us, that cries aloud, "It is your turn—endure." Play your part. As they endured before you, so now, close up the ranks—be patient and strong as they were. Since Christ, this world of pain is no accident untoward or sinister, but a lawful department of life, with experiences, interests, adventures, hopes, delights, secrets of its own. These are all thrown open to us as we pass within the gates—things that we could never learn or know or see, so long as we were well. God help you to walk through this world now opened to you, as through a kingdom, regal, royal, and wide and glorious.

*Canon Scott Holland.*
"*Extract from Letter to G. J. Romanes.*"

Suffering at first isolates us; but afterwards it links us in the closest bonds with all who are sitting on the hard benches of the school of sorrow. We learn to comfort them with the comfort with which we have ourselvees been comforted of God. The water streams from the smitten rock. The flower springs from the dead seed. The crystal river flows from the melting glacier. The bright gold emerges from the dark mine and the cleansing fires.

*Rev. F. B. Meyer.*

Bright, radiant, victorious faith is essential in one who would give real consolation. . . . Mere pity alone leaves the heart weaker than before. Wise and true comfort must give something that shall prove strength and inspiration to the fainting spirit, and help it to rise again. It should be like the wine which angels of mercy pour into the lips of the wounded on the fields of battle to revive them.

*J. R. Miller, D.D.* "*Silent Times.*"

### Notes and Queries.

**The Editor is always willing to answer in this column, without any fee, all reasonable questions, as soon as possible.**
**But the following rules must be carefully observed:—**
**1. Every communication must be accompanied by the name and address of the writer.**
**2. The question must always bear upon nursing, directly or indirectly.**
**If an answer is required by letter a fee of half-a-crown must be enclosed with the note containing the inquiry.**

*Heterogenetic Infection.*

(202) 1. Is there any fear of heterogenetic infection arising amongst patients in a lying-in ward where a woman has been admitted suffering from lupus in an advanced stage with necrosed bone? 2. If so, where does the danger lie? 3. What would be the correct method of feeding her infant?—*Perplexed.*

1. Presuming that by the term heterogenetic infection "Perplexed" means an infection arising from some source outside the patient, which is what ordinary people understand by the simple word infection, without the addition of the adjective, it is obvious that, other things being equal, there is more likelihood of its arising when a source from which it might be derived is present than when there is no such source within a ward. 2. The modes by which infective material may gain access to a patient's body are manifold, but in the hypothetical case suggested the nurses' hands would be the most likely route. 3. This must depend upon circumstances. If the patient is in a hospital, which here would seem to be the case, the medical officer in charge will decide.

*Mental Derangement.*

(203) Can you give me the name of any institutions where a strong able-bodied woman of 43 could be received for £25 a year?—*W. H.*

Could you tell me of a home where a coachman's daughter, aged 12 years, feeble-minded, could be received?—*Nurse B.*

Could you advise me where to place a little idiot boy of eight?—*A. W.*

The Secretary, The National Association for Promoting the Welfare of the Feeble-minded, 53 Victoria Street, S.W.; and the Secretary of the Metropolitan Asylums Board Offices, Victoria Embankment, E.C., would be able to advise you.

*Epileptic.*

(204) Will you kindly tell me of a home where an epileptic boy of nineteen could be received? He is amiable and intelligent, and until recently has been able to earn his living as an errand boy.—*C. C. P.*

Apply the Secretary, The Epileptic Colony, Chalfont St. Peter's (office, 12 Buckingham Street, W.C.), and the Secretary the Home for Epileptics, Maghull, near Liverpool (office, 2 Exchange Street East, Liverpool).

*Liverpool.*

(205) 1. Will you kindly let me know of one or two of the best General and Fever Hospitals in Liverpool. 2. Is the certificate from a Liverpool hospital equal in value to one obtained in a London Union Infirmary?—*Probationer Z.*

The Mill Road Infirmary, the Liverpool Northern Hospital, the Liverpool Royal Infirmary, the Royal Southern Hospital, and the Workhouse Infirmary, Brownlow Hill, are all training schools recognised by the Local Government Board, and their certificates are equal to those gained in London Union Infirmaries.

Can you give me the address of Fever and General Hospitals in Liverpool where a nurse with two years' training might obtain employment and a salary of not less than £18 a year?—*Nurse W.*

You will find a list of hospitals for infectious disease on p. 508 in "Burdett's Hospitals and Charities." We cannot say what vacancies there may be in the nursing staff, nor whether they will accept a two-years' certificate.

*Light Nursing.*

(206) I am a nurse in a special hospital for the Paralysed; after fourteen months' training, however, I am obliged to leave on account of my health. Could you tell me of a hospital where there is no heavy lifting?—*Anxious One.*

We can only suggest children's hospitals and convalescent homes.

*Wanted, Probationers.*

(207) Will you please recommend this home to paying probationers who wish to be well trained; and will you also send me the names of any people who can pay for being nursed, as I take in invalids.—*Matron.*

We cannot recommend private institutions either to probationers or patients.

*Exam. in Mental Nursing.*

(208) Will you kindly tell me if I am eligible for an examination in asylum work. I am assistant matron here?—*Dela.*

The asylum at which you are prepares candidates for the Medico-Psycological Examination. Consult your medical officer.

*Unfit Probationer.*

(209) I obtained a post in a small cottage hospital, but the air did not suit me, and I was obliged to return home at the end of my mouth's trial. I then became probationer in a large sanatorium, but, unfortunately, six days afterwards I again took ill. Do you think I should try again?—*Gordon.*

If you are anxious to try again it would seem that the question is whether you are strong enough. As to this consult a doctor.

**Standard Books of Reference.**

**"The Nursing Profession: How and Where to Train." 2s. net; post free 2s. 4d.**
**"Burdett's Official Nursing Directory." 3s. net.; post free, 3s. 4d.**
**"Burdett's Hospitals and Charities." 5s.**
**"The Nurses' Dictionary of Medical Terms." 2s.**
**"Burdett's Series of Nursing Text-Books." 1s. each.**
**"A Handbook for Nurses." (Illustrated.) 5s.**
**"Nursing: Its Theory and Practice." New Edition. 3s. 6d.**
**"Helps in Sickness and to Health." Fifteenth Thousand. 5s.**
**"The Physiological Feeding of Infants." 1s.**
**"The Physiological Nursery Chart." 1s.; post free, 1s. 3d.**
**"Hospital Expenditure: The Commissariat." 2s. 6d.**
**All these are published by THE SCIENTIFIC PRESS, Ltd., and may be obtained through any bookseller or direct from the publishers, 28 and 29 Southampton Street, London, W.C.**

